# Complete genome sequence of the industrial bacterium *Bacillus licheniformis *and comparisons with closely related *Bacillus *species

**DOI:** 10.1186/gb-2004-5-10-r77

**Published:** 2004-09-13

**Authors:** Michael W Rey, Preethi Ramaiya, Beth A Nelson, Shari D Brody-Karpin, Elizabeth J Zaretsky, Maria Tang, Alfredo Lopez de Leon, Henry Xiang, Veronica Gusti, Ib Groth Clausen, Peter B Olsen, Michael D Rasmussen, Jens T Andersen, Per L Jørgensen, Thomas S Larsen, Alexei Sorokin, Alexander Bolotin, Alla Lapidus, Nathalie Galleron, S Dusko Ehrlich, Randy M Berka

**Affiliations:** 1 Novozymes Biotech Inc, 1445 Drew Ave, Davis, CA 95616, USA; 2 Novozymes A/S, Bagsværd, DK-2880, Denmark; 3 Institut National de la Recherche Agronomique, Paris Cedex 75007, France; 4 AstraZeneca International, Lund SE221 87, Sweden; 5 Joint Genome Institute, Walnut Creek, CA 94598, USA

## Abstract

**Background:**

*Bacillus licheniformis *is a Gram-positive, spore-forming soil bacterium that is used in the biotechnology industry to manufacture enzymes, antibiotics, biochemicals and consumer products. This species is closely related to the well studied model organism *Bacillus subtilis*, and produces an assortment of extracellular enzymes that may contribute to nutrient cycling in nature.

**Results:**

We determined the complete nucleotide sequence of the *B. licheniformis *ATCC 14580 genome which comprises a circular chromosome of 4,222,336 base-pairs (bp) containing 4,208 predicted protein-coding genes with an average size of 873 bp, seven rRNA operons, and 72 tRNA genes. The *B. licheniformis *chromosome contains large regions that are colinear with the genomes of *B. subtilis *and *Bacillus halodurans*, and approximately 80% of the predicted *B. licheniformis *coding sequences have *B. subtilis *orthologs.

**Conclusions:**

Despite the unmistakable organizational similarities between the *B. licheniformis *and *B. subtilis *genomes, there are notable differences in the numbers and locations of prophages, transposable elements and a number of extracellular enzymes and secondary metabolic pathway operons that distinguish these species. Differences include a region of more than 80 kilobases (kb) that comprises a cluster of polyketide synthase genes and a second operon of 38 kb encoding plipastatin synthase enzymes that are absent in the *B. licheniformis *genome. The availability of a completed genome sequence for *B. licheniformis *should facilitate the design and construction of improved industrial strains and allow for comparative genomics and evolutionary studies within this group of Bacillaceae.

## Abstract 2

The complete sequence of the *Bacillus licheniformis *ATCC 14580 genome was determined, revealing 4,208 predicted protein-coding genes, 7 rRNA operons and 72 tRNA genes.

## Background

*Bacillus licheniformis *is a Gram-positive, spore-forming bacterium widely distributed as a saprophytic organism in the environment. This species is a close relative of *Bacillus subtilis*, an organism that is second only to *Escherichia coli *in the level of detail at which it has been studied. Unlike most other bacilli, which are predominantly aerobic, *B. licheniformis *is a facultative anaerobe, which may allow it to grow in additional ecological niches. Certain *B. licheniformis *isolates are capable of denitrification; the relevance of this characteristic to environmental denitrification may be small, however, as the species generally persists in soil as endospores [1].

There are numerous commercial and agricultural uses for *B. licheniformis *and its extracellular products. The species has been used for decades in the manufacture of industrial enzymes including several proteases, α-amylase, penicillinase, pentosanase, cycloglucosyltransferase, β-mannanase and several pectinolytic enzymes. The proteases from *B. licheniformis *are used in the detergent industry as well as for dehairing and bating of leather [2,3]. Amylases from *B. licheniformis *are deployed for the hydrolysis of starch, desizing of textiles and sizing of paper [3]. Specific *B. licheniformis *strains are also used to produce peptide antibiotics such as bacitracin and proticin in addition to a number of specialty chemicals such as citric acid, inosine, inosinic acid and poly-γ-glutamic acid [4]. Some *B. licheniformis *isolates can mitigate the affects of fungal pathogens on maize, grasses and vegetable crops [5]. As an endospore-forming bacterium, the ability of the organism to survive under unfavorable environmental conditions may enhance its potential as a natural biocontrol agent.

*B. licheniformis *can be differentiated from other bacilli on the basis of metabolic and physiological tests [6,7]; however, biochemical and phenotypic characteristics may be ambiguous among closely related species. Recent taxonomic studies indicate that *B. licheniformis *is closely related to *B. subtilis *and *Bacillus amyloliquefaciens *on the basis of comparisons of 16S rDNA and 16S-23S internal transcribed spacer (ITS) nucleotide sequences [8]. Lapidus *et al. *[9] recently constructed a physical map of the *B. licheniformis *chromosome using a PCR approach, and established a number of regions of colinearity where gene content and organization were conserved with the *B. subtilis *genome.

Given that *B. licheniformis *is an industrial organism used for the manufacture of enzymes, antibiotics, and chemicals, important in nutrient cycling in the environment, and a species that is taxonomically related to *B. subtilis*, perhaps the best studied of all Gram-positive bacteria, we derived the complete nucleotide sequence of the *B. licheniformis *type strain (ATCC 14580) genome. With this data in hand, functional and comparative genomics studies can be initiated that may ultimately lead to new strategies for improving industrial strains as well as better understanding of genome evolution among the species within the *subtilis-licheniformis *group.

## Results and discussion

### General features of the *B. licheniformis *genome

**Figure 1:**
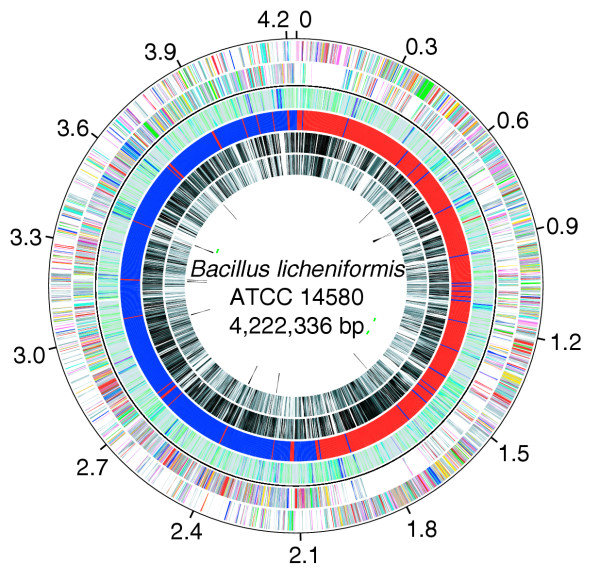
Circular representation of the *B. licheniformis *ATCC 14580 chromosome. Circles are numbered from 1 (outermost) to 7 (innermost). Circles 1 and 2 show the locations of predicted CDSs on the + and - strands, respectively; circle 3, %G+C; circle 4, GC skew ((G-C/G+C)); circle 5, homology with *B. subtilis *168; circle 6, homology with *B. halodurans*; circle 7 shows positions of nine copies of insertion sequence element *IS3Bli1 *and a putative transposase gene; small green bars inside circle 7 denote the positions of possible prophage elements.

The genome of *B. licheniformis *ATCC 14580 consists of a circular chromosome of 4,222,336 base-pairs (bp) with an average G+C content of 46.2% (Table 1). No plasmids were found during the genome analysis, and none were found by agarose gel electrophoresis (data not shown). Using a combination of several gene-finding programs and manual inspection, 4,208 protein-coding sequences (CDSs) were predicted. These CDSs constitute 87% of the genome and have an average length of 873 bp (ranging from 78 to 10,767 bp). They are oriented on the chromosome primarily in the direction of replication (Figure 1) with 74.4% of the genes on the leading strand and 25.6% on the lagging strand. Among the 4,208 protein coding genes, 3,948 (94%) had significant similarity to proteins in PIR, 3,187 (76%) of these gene models contain Interpro motifs, and 2,895 (69%) contain protein motifs found in PFAM. The number of hypothetical and conserved hypothetical proteins in the *B. licheniformis *genome with hits in the PIR database was 1,318 (212 conserved hypothetical proteins). Among the list of hypothetical and conserved hypothetical gene products, 683 (52%) have protein motifs contained in PFAM (148 conserved hypothetical proteins). There are 72 tRNA genes representing all 20 amino acids and seven rRNA operons.

The likely origin of replication (Figure 1) was identified by similarities to several features of the corresponding regions in *B. subtilis *and other bacteria. These included co-localization of four genes (*rpmH*, *dnaA*, *dnaN*, and *recF*) near the origin, GC nucleotide skew ((G-C)/(G+C)) analysis, and the presence of multiple *dnaA*-boxes and AT-rich sequences immediately upstream of the *dnaA *gene [10-12]. On the basis of these observations we assigned a cytosine residue of the *Bst*BI restriction site between the *rpmH *and *dnaA *genes to be the first nucleotide of the *B. licheniformis *genome. The replication termination site was localized near 2.02 megabases (Mb) by GC skew analysis. This region lies roughly opposite the origin of replication (Figure 1). Unlike *B. subtilis*, there was no apparent gene encoding a replication terminator protein (*rtp*) in *B. licheniformis*. The *Bacillus halodurans *genome also lacks an obvious *rtp *function [13]; therefore, it seems likely that *B. subtilis *acquired the *rtp *gene following its divergence from *B. halodurans *and *B. licheniformis*.

### Transposable elements, prophages and atypical regions

**Figure 2:**
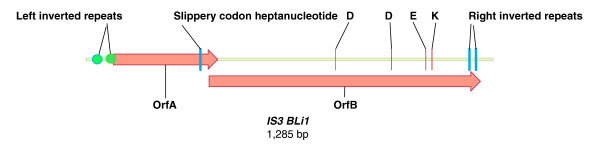
Schematic map of the insertion sequence *IS3Bli1*. Nine identical copies of this 1,285-bp element were found in the genome of *B. licheniformis *ATCC 14580. Features of the *IS3Bli1 *element are summarized in the text.

The genome of *B. licheniformis *ATCC 14580 contains nine identical copies of a 1,285 bp insertion sequence element termed *IS3Bli1 *[9]. This sequence shares a number of features with other *IS3 *family elements [9] including direct repeats of 3-5 bp, a 10-bp left inverted repeat, and a 9 bp right inverted repeat (Figure 2). *IS3Bli1 *encodes two predicted overlapping CDSs, designated *orfA *and *orfB *in relative translational reading frames of 0 and -1. The presence of a 'slippery heptamer' motif, AAAAAAG, before the stop codon in *orfA *may indicate that programmed translational frameshifting occurs between these two coding sequences, resulting in a single gene product [14]. The *orfB *gene product harbors the DD(35)E(7)K motif, a highly conserved pattern among insertion sequences. Eight of these *IS3Bli1 *elements lie in intergenic regions, and one interrupts the *comP *gene as noted previously [9]. In addition to these insertion sequences, the genome encodes a putative transposase that is most closely related (E = 1.8 × 10^-11^) to one identified in the *Thermoanaerobacter tengcongensis *genome [15]; however, similar transposase genes are also found in the chromosomes of *B. halodurans *[13], *Oceanobacillus iheyensis *[16], *Streptococcus agalactiae *[17] and *Streptococcus pyogenes *[18].

**Figure 3:**
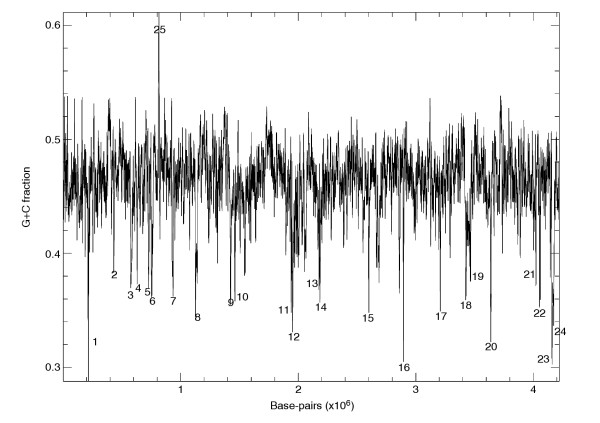
Isochore plot of the *B. licheniformis *ATCC 14580 genome showing G+C content as a function of position. AT-rich peaks (numbered 1-24) are marked on the plot, and a single island that is atypically GC-rich is indicated by number 25. Table 2 lists the specific chromosomal features represented by each numbered peak.

The presence of several bacteriophage lysogens or prophage-like elements was revealed by Smith-Waterman comparisons to other bacterial genomes and by their AT-rich signatures (Figure 3, Table 2). Prophage sequences, designated NZP1 and NZP3 (similar to *B. subtilis *prophages PBSX and φ-105), were discovered by noting the presence of nearby genes that code for the large subunit of terminase, a signature protein that is highly conserved among prophages [19]. Interestingly, a terminase gene was not observed in the third putative prophage, termed NZP2 (similarity to *B. subtilis *phage SPP1); however, its absence may be the result of genome deterioration during evolution. Interestingly, we observed that regions in which the G+C content is less than 39% usually encoded proteins that have no *B. subtilis *ortholog and share identity only with hypothetical and conserved hypothetical genes. Two of these AT-rich segments correspond to the NZP2 and NZP3 prophages.

An isochore plot (Figure 3) also revealed the presence of a region with an atypically high (62%) G+C content. This segment contains two hypothetical genes whose sizes (3,831 and 2,865 bp) greatly exceed the size of an average CDS in *B. licheniformis*. The first gene encodes a protein of 1,277 amino acids for which Interpro predicts 16 collagen triple-helix repeats, and the amino acid pattern TGATGPT is repeated 75 times within the polypeptide. The second CDS is smaller, and encodes a protein with 11 collagen triple-helix repeats; the same TGATGPT motif recurs 56 times. The primary translation products from these genes do not contain canonical signal peptides for secretion, and they do not contain motifs for the twin-arginine or sortase-mediated translocation pathways. Therefore, it is not likely that they are exported to the cell surface or the extracellular medium. Interestingly, the chromosomal region (19 kb) adjacent to these genes is clearly non-colinear with the *B. subtilis *genome, and virtually all of the predicted genes encode hypothetical or conserved hypothetical proteins. There are a number of bacterial proteins listed in PIR that also contain collagen triple-helix repeat regions, including two from *Mesorhizobium loti *(accession numbers NF00607049 and NF00607035) and three from *B. cereus *(accession numbers NF01692528, NF01269899 and NF01694666). These putative orthologs share 53-76% amino-acid sequence identity with their counterparts in *B. licheniformis*, and their functions are unknown.

### Extracellular enzymes and metabolic activities

In the *Bacillus licheniformis *genome, 689 of the 4,208 gene models have signal peptides forecast by SignalP [20]. Of these, 309 have no transmembrane domain predicted by TMHMM [21] and 134 are hypothetical or conserved hypothetical genes. Based on a manual examination of the remaining 175 genes, at least 82 are likely to encode secreted proteins and enzymes. Moreover, there are 27 predicted extracellular proteins encoded by the *B. licheniformis *ATCC 14580 genome that are not found in *B. subtilis *168. In accordance with its saprophytic lifestyle, the secretome of *B. licheniformis *encodes numerous secreted enzymes that hydrolyze polysaccharides, proteins, lipids and other nutrients.

Cellulose is the most abundant polysaccharide on Earth, and microorganisms that hydrolyze cellulose contribute to the global carbon cycle. Interestingly, two gene clusters involved in cellulose degradation and utilization were discovered in *B. licheniformis*, and there are no counterparts in *B. subtilis *168. The enzymes encoded by the first gene cluster include two putative endoglucanases belonging to glycoside hydrolase families GH9 and GH5, a probable cellulose-1,4-β-cellobiosidase of family GH48, and a putative β-mannanase of family GH5. The β-mannanase (GH5) and endoglucanase (GH9) both harbor carbohydrate-binding motifs. With the exception of the cellulose-1,4-β-cellobiosidase (GH48), all of the gene products encoded in this cluster have secretory signal peptides, and all have homologs in *Bacillus *species other than *B. subtilis*. The overall G+C content of this cluster (48%) does not appear to differ appreciably from that of the genome average (46%). The second gene cluster encodes a putative β-glucosidase (GH1) and three components of a cellobiose-specific PTS transport complex. A second β-glucosidase (GH3) gene is present at an unlinked locus in the genome. Collectively, the genes in these two clusters should enable *B. licheniformis *to utilize cellulose as a carbon and energy source, converting it to cellobiose and ultimately glucose. In this regard, we have confirmed that *B. licheniformis *ATCC 14580 is capable of growth on carboxymethyl cellulose as a sole carbon source (not shown). The chromosome of *B. licheniformis *ATCC 14580 encodes a number of additional carbohydrase activities that may allow the organism to grow on a broad range of polysaccharides. These include xylanase, endo-arabinase and pectate lyase that may be involved in degradation of hemicellulose, α-amylase and α-glucosidase for starch hydrolysis, chitinases for the breakdown of chitooligosaccharides from fungi and insects, and levanase for utilization of β-D-fructans (levans). Several of these activities are marketed as industrial enzymes.

Saprophytic organisms must utilize a variety of nitrogenous compounds as nutrients for growth and metabolism. On the basis of the information encoded in its genome, *B. licheniformis *ATCC 14580 possesses the ability to acquire nitrogen from exogenous proteins, peptides, amino acids, ammonia, nitrate and nitrite. Like *B. subtilis*, the repertoire of extracellular proteases produced by *B. licheniformis *includes serine proteases (*aprE*, *epr*, *vpr*), metalloprotease (*mpr*), and an assortment of endo- and exopeptidases (*yjbG*, *ydiC*, *gcp*, *ykvY*, *ampS*, *bpr *(two copies), *yfxM*, *yuiE*, *yusX*, *ywaD*, *pepT*). However, *B. licheniformis *also has the capacity to produce a number of additional proteases and peptidases that are not encoded in the *B. subtilis *genome. These include a clostripain-like protease, a zinc-metallopeptidase, a probable glutamyl endopeptidase, an aminopeptidase C homolog, two putative dipeptidases and a zinc-carboxypeptidase.

*B. licheniformis *also has the ability to utilize amino and imino nitrogen from arginine, asparagine and glutamine via arginine deiminase, arginase, asparaginase and glutaminase activities. Interestingly, there appear to be two genes each for arginase, asparaginase and glutaminase. Presumably, the arginine deiminase activity is expressed during anaerobic growth on arginine, whereas the arginase activities are predominant during aerobic growth. The occurrence of putative arginase genes is somewhat of an enigma in *B. licheniformis*, because there are no genes encoding urease activity for the hydrolysis of urea that is generated by the arginase reaction. In addition to the absence of urease gene homologs (*ureABC*) in *B. licheniformis*, the glutamine ABC transporters (*glnH*, *glnM*, *glnP*, *glnQ *gene products) are also lacking.

It appears that nitrogen assimilation and transport pathways may be coordinated similarly in *B. licheniformis *and *B. subtilis *owing to the presence of key genes such as *glnA*, *glnR*, *tnrA *and *nrgA *in both species. Likewise, the pathways for nitrate/nitrite transport and metabolism in *B. licheniformis *appear to be analogous to the corresponding pathways in *B. subtilis *as suggested by the presence of *nasABC *(nitrate transport), *narGHIJ *(respiratory nitrate reductase), and *nasDEF *(NADH-dependent nitrite reductase) genes. Unlike *B. subtilis*, *B. licheniformis *evidently possesses the capability for anaerobic respiration using nitric oxide reductase. Moreover, the gene encoding this activity lies in a cluster that includes CDSs for *narK *(nitrite extrusion protein), two putative *fnr *proteins (transcriptional regulators of anaerobic growth), and a *dnrN*-like gene product (nitric oxide-dependent regulator). These observations are consistent with previous findings that certain *B. licheniformis *isolates are capable of denitrification [22]. While denitrification is a process of major ecological importance, the contribution of *B. licheniformis *may be small as the species exists predominantly as endospores in soil [1].

Microbial D-hydantoinase enzymes have been applied to the industrial production of optically pure D-amino acids for synthesis of antibiotics, pesticides, sweeteners and therapeutic amino acids [23]. This enzyme catalyzes the hydrolysis of cyclic ureides such as dihydropyrimidines and 5-monosubstituted hydantoins to *N*-carbamoyl amino acids. Hydantoinase activities have been detected in a variety of bacterial genera, and a cluster of six genes in *B. licheniformis *appears to confer a similar capability. This gene cluster encodes *N*-methylhydantoinase (ATP-hydrolyzing), hydantoin utilization proteins A and B (*hyuAB *homologs), a possible transcriptional regulator (TetR/AcrR family), a putative pyrimidine permease, and a hypothetical protein that contains an Interpro domain (IPR004399) for phosphomethylpyrimidine kinase.

### Protein export, sporulation and competence pathways

Kunst *et al*. [10] listed 18 genes that have a major role in the secretion of extracellular enzymes by the classical (Sec) pathway in *B. subtilis *168. This list includes several chaperonins, signal peptidases, components of the signal recognition particle and protein translocase complexes. All members of this list have *B. licheniformis *counterparts. In addition to the Sec pathway, some *B. subtilis *proteins are directed into the twin-arginine (Tat) export pathway, possibly in a Sec-independent manner. Curiously, the *B. licheniformis *genome encodes three *tat *gene orthologs (*tatAY*, *tatCD*, and *tatCY*), but two others (*tatAC *and *tatAD*) are conspicuously absent. Furthermore, specific proteins may be exported to the cell surface via lipoprotein signal peptides or sortase factors. Lipoprotein signal peptides are cleaved with a specific signal peptidase (Lsp) encoded by the *lspA *gene in *B. subtilis*. An *lspA *homolog can be found in *B. licheniformis *as well, suggesting that this species may possess the ability to export lipoproteins via a similar mechanism. Lastly, surface proteins in Gram-positive bacteria are frequently attached to the cell wall by sortase enzymes, and genome analyses have revealed that more than one sortase is often produced by a given species. In this regard, three possible sortase gene homologs were detected in the genome of *B. licheniformis *ATCC 14580. Together these observations suggest that the central features of the protein export machinery are principally conserved in *B. subtilis *and *B. licheniformis*.

From the list of 139 sporulation genes tabulated by Kunst *et al*. [10], all but six have obvious counterparts in *B. licheniformis*. These six exceptions (*spsABCEFG*) comprise an operon involved in synthesis of a spore coat polysaccharide in *B. subtilis*. In addition, the response regulator gene family (*phrACEFGI*) appears to have a low level of sequence conservation between *B. subtilis *and *B. licheniformis*.

Natural competence (the ability to take up and process exogenous DNA in specific growth conditions) is a feature of few *B. licheniformis *strains [24]. The reasons for variability in competence phenotype have not been explored at the genetic level, but the genome data offer several possible explanations. Although the *B. licheniformis *genome encodes all of the late competence functions ascribed in *B. subtilis *(for example, *comC*, *comEFG *operons, *comK*, *mecA*), it lacks an obvious *comS *gene, and the *comP *gene is punctuated by an insertion sequence element (*IS3Bli1*), suggesting that the early stages of competence development have been pre-empted in *B. licheniformis *ATCC 14580. Whether these early functions can be restored by introducing the corresponding genes from *B. subtilis *is unknown. In addition to an apparent deficiency in DNA uptake, two type I restriction-modification systems were discovered that may also contribute to diminished transformation efficiencies. These are distinct from the *ydiOPS *genes of *B. subtilis*, and could participate in degradation of improperly modified DNA from heterologous hosts used during construction of recombinant expression vectors. Each of these loci in *B. licheniformis *(designated as *BliI *and *BliII*) encode putative HsdS, HsdM and HsdR subunits that share significant amino-acid sequence identity to type I restriction-modification proteins in other bacteria. Curiously, the G+C-content for these loci (37%) is substantially lower than the overall genome average (46%) which may hint that they are the result of gene acquisitions. Lastly, the synthesis of a glutamyl polypeptide capsule has also been implicated as a potential barrier to transformation of *B. licheniformis *strains [25]. While laboratory strains of *B. subtilis *usually do not produce significant capsular material, the genome sequence of *B. subtilis *168 indicates that they may harbor the genes required for synthesis of polyglutamic acid. In contrast, many *B. licheniformis *isolates produce copious amounts of capsular material, giving rise to colonies with a wet or slimy appearance. Six genes were predicted (*ywtABDEF *and *ywsC *orthologs) that may be involved in the synthesis of polyglutamic acid capsular material in *B. licheniformis*.

### Antibiotics, secondary metabolites and siderophores

Bacitracin is a cyclic peptide antibiotic that is synthesized non-ribosomally by some *B. licheniformis *isolates [26]. While there is variation in the prevalence of bacitracin synthase genes among laboratory strains of this species, one study suggested that up to 50% may harbor the *bac *operon [27]. Interestingly, the *bac *operon is not present in the type strain (ATCC 14580) genome. Seemingly, the only non-ribosomal peptide synthase operon encoded by the *B. licheniformis *type strain genome is that responsible for lichenysin biosynthesis. Lichenysin structurally resembles surfactin from *B. subtilis *[28], and their respective biosynthetic operons are highly similar. Surprisingly, we found no *B. licheniformis *counterparts for the *pps *(plipastatin synthase) and polyketide synthase (*pks*) operons of *B. subtilis*. Collectively, these two regions represent sizeable portions (80 kb and 38 kb, respectively) of the chromosome in *B. subtilis*, although they are reportedly dispensable [29].

Unexpectedly, a cluster of 11 genes was found encoding a lantibiotic, with its associated modification and transport functions. We designated this peptide of 75 amino acids as lichenicidin, and its closest homolog is mersacidin from *Bacillus *sp. strain HIL-Y85/54728 [30]. Lantibiotics are ribosomally synthesized peptides that are modified post-translationally so that the final molecules contain rare thioether amino acids such as lanthionine and/or methyl-lanthionine [31]. Like mersacidin, lichenicidin appears to be a type B lantibiotic, comprising a rigid globular peptide with no net charge (7 acidic residues, 7 basic residues) and a leader peptide with a conserved double glycine cleavage site (GG-type leader peptide). These antimicrobial compounds have attracted much attention in recent years as models for the design and genetic engineering of improved antimicrobial agents [32]. However, since several post-translational modifications and product-specific export functions are required, a dedicated expression system is a prerequisite to provide all the factors necessary to synthesize, modify and transport the lantibiotic peptide. With its history of use in industrial microbiology, *B. licheniformis *may be an attractive candidate for the development of such an expression system.

Like *B. subtilis *168, the *B. licheniformis *ATCC 14580 chromosome harbors a siderophore biosynthesis gene cluster (*dhbABCEF*), and the organization of the cluster is similar to the corresponding chromosomal segment in *B. subtilis*. In addition, the *B. licheniformis *genome contains a second gene cluster of four genes (*iucABCD*) that show significant similarity to proteins involved in aerobactin biosynthesis in *E. coli*. Surprisingly, a gene encoding the receptor protein (*iutA *homolog) was not found in *B. licheniformis*. The *B. halodurans *genome also contains genes that are homologous to *iucABCD*, but like *B. licheniformis*, no *iutA *homolog could be found using BLAST or Smith-Waterman searches.

### Comparison of the *B. licheniformis *genome with those of other bacilli

**Figure 4:**
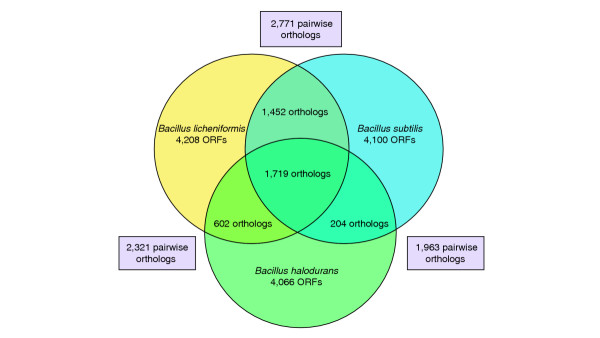
Venn diagram comparing the orthologous gene complements of *B. licheniformis *ATCC 14580, *B. subtilis *168 and *B. halodurans *C-125. Numbers in purple boxes indicate the number of pairwise orthologs between adjacent species (BLAST threshold E = 1 × 10^-5^). Numbers in the outer circles represent the total number of CDSs predicted in each genome, numbers in areas of overlap indicate the number of orthologs predicted by reciprocal BLASTP analysis (threshold E = 1 × 10^-5^), and the number in the center gives the number of orthologous sequences common to all three genomes.

The *B. licheniformis *ATCC 14580 gene models were compared to the list of essential genes in *B. subtilis *[33]. Predictably, all of the essential genes in *B. subtilis *have orthologs in *B. licheniformis*, and most are present in a wide range of bacterial taxa. In pairwise BLAST comparisons, 66% of the predicted *B. licheniformis *genes have orthologs in *B. subtilis*, and 55% of the gene models are represented by orthologous sequences in *B. halodurans *(E-value threshold of 1 × 10^-5^; Figure 4). Using a reciprocal BLASTP analysis we found 1,719 orthologs that are common to all three species (E-value threshold of 1 × 10^-5^).

**Figure 5:**
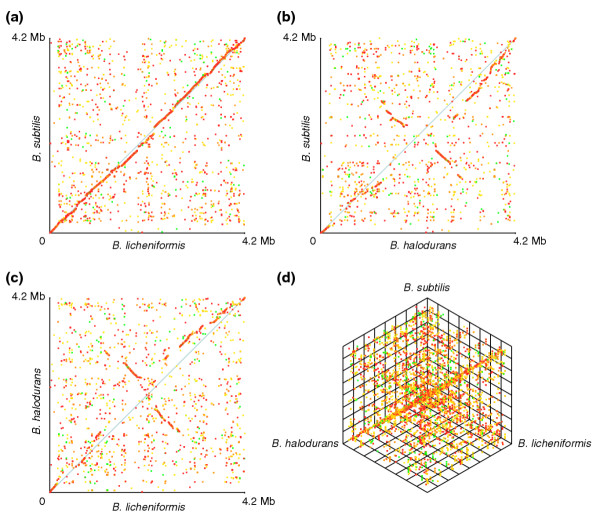
Two- and three-dimensional similarity plots comparing the distribution of orthologs on the chromosomes of *B. licheniformis*, *B. subtilis *and *B. halodurans*. BLAST scores were generated and dots were positioned according to the locations in the genome where orthologs exist in order to view possible regions of possible colinearity. The minimum BLAST expectancy score for each dot in this example was 1 × 10^-50^. **(a) **The plot for *B. licheniformis *versus *B. subtilis*; **(b) ***B. halodurans *versus *B. subtilis*; **(c) ***B. licheniformis *versus *B. halodurans*; **(d) **a three-dimensional scatter plot comparing the distribution of orthologs among all three species. Dots located on the diagonal are indicative of conserved location of orthologous genes between species, whereas a line of dots that lie perpendicular to the diagonal suggests an inversion of a genomic segment between species.

As noted by Lapidus *et al. *[9], there are broad regions of colinearity between the genomes of *B. licheniformis *and *B. subtilis *(Figure 5). Less conservation of genome organization exists between *B. licheniformis *and *B. halodurans*, and substantial genomic segments have been inverted in *B. halodurans *with respect to *B. licheniformis *and *B. subtilis*. These observations clearly support previous hypotheses [8] that *B. subtilis *and *B. licheniformis *are phylogenetically and evolutionarily closer to each other than to *B. halodurans*.

## Conclusions

In comparisons of shared regions, the genomes of *B. licheniformis *ATCC 14580 and *B. subtilis *168 are approximately 84.6% identical at the nucleotide level and show extensive organizational similarity. Accordingly, their genome sequences represent potentially useful instruments for comparative and evolutionary studies among species within the *subtilis-licheniformis *group, and they may offer new information regarding the evolution and ecology of these closely related species.

Despite the broad colinearity of *B. licheniformis *and *B. subtilis *genomes, there are local regions that are individually unique. These include chromosome segments that comprise prophage and insertion sequence elements, DNA restriction-modification systems, antibiotic synthases, and a number of extracellular enzymes and metabolic activities that are not present in *B. subtilis*. It is tempting to speculate that the presence of these genes forecasts the ability of *B. licheniformis *to grow on an expanded array of substrates and/or in additional ecological niches compared to *B. subtilis*. Together, the similarities and differences may hint at overlapping but non-identical environmental niches for these taxa.

The *subtilis-licheniformis *group of bacilli includes many strains that are used to manufacture industrial enzymes, antibiotics and biochemicals. The availability of a complete genome from *B. licheniformis *should permit a thorough comparison of the biochemical pathways and regulatory networks in *B. subtilis *and *B. licheniformis*, thereby offering new opportunities and strategies for improvement of industrial strains. When considering the safety of *B. licheniformis *as an industrial organism it should be noted that the species is considered neither a human pathogen nor a toxigenic microorganism [34]; however, there are reports in the literature implicating it as a causal agent of food poisoning. In these isolated cases, specific strains were shown to produce a toxin similar to cereulide, the emetic toxin of *B. cereus *[35]. Cereulide is a cyclic depsipeptide synthesized non-ribosomally [36]. Importantly, the only non-ribosomal peptide synthase genes found in the *B. licheniformis *ATCC 14580 genome are those that involved in synthesis of lichenysin. Similarly, we detected no homologs of the *B. cereus *hemolytic and non-hemolytic enterotoxins (Swiss-Prot accession numbers P80567, P80568, P80172, and P81242).

In a comparison of the genotypic and phenotypic characteristics among 182 soil isolates of *B. licheniformis*, Manachini *et al. *[37] observed that while this bacterial species appears to be phenotypically homogeneous, clear genotypic differences are evident between isolates. They postulated the existence of three genomovars for *B. licheniformis*. Similarly, De Clerck and De Vos [38] proposed that this species consists of two lineages that can be distinguished using several molecular genotyping methods. The genome sequence data presented in this work should provide a solid foundation on which to conduct future studies to elucidate the genotypic variation among *B. licheniformis *isolates.

## Materials and methods

### Shotgun DNA sequencing and genome assembly

The genome of *B. licheniformis *ATCC 14580 was sequenced by a combination of the whole-genome shotgun method [39] and fosmid end sequencing [40]. Plasmid libraries were constructed using randomly sheared and *Mbo*I-digested genomic DNA that was enriched for fragments of 2-3 kb by preparative agarose gel electrophoresis. Approximately 49,000 random clones were sequenced using dye-terminator chemistry (Applied Biosystems) with ABI 377 and ABI 3700 automated sequencers yielding approximately 6× coverage of the genome. A combination of methods was used for gap closure, including sequencing on fosmids [40] and primer-walking on selected clones and PCR-amplified DNA fragments. We also incorporated data from both ends of approximately 1,975 fosmid clones with an average insert size of 40 kb to aid in validating the final assembly. In total, the number of input reads was 62,685, with 78.6% of these incorporated into the assembled genome sequence. Individual nucleotides were called using TraceTuner 2.0 (Paracel), and sequence reads were assembled into contigs using the Paracel Genome Assembler using optimized parameters and the quality score set to >20. Phrap, Crossmatch and Consed were used for sequence finishing [41].

### Prediction and annotation of CDSs

Protein-coding regions in the assembled genome sequence were identified using a combination of previously described software tools including EasyGene [42], Glimmer [43] and FrameD [44]. EasyGene was used as the primary gene finder in these studies. It searches for protein matches in the raw genome data to derive a good training set, and an HMM with states for coding regions as well as ribosome-binding sites (RBSs) is estimated from the dataset. This HMM is used to score all the predicted CDSs in the genome, and the score is converted to a measure of significance (R-value) which is the expected number of CDSs that would be predicted in 1 Mb of random DNA. Gene models with R-values lower than 10 and a log-odds score of greater than -10 were included/considered significant. The principal advantage of this significance measure is that it properly takes into account the length distribution of random CDSs. EasyGene has been shown to match or exceed other gene finders currently available [42]. Glimmer was used as a secondary gene finder to aid in identification of small genes (< 100 bp) that were sometimes missed by EasyGene. Glimmer models were post-processed with RBSFINDER [45] to pinpoint the positions of start codons by searching for consensus Shine-Dalgarno sequences. According to the RBS states in the EasyGene HMM model, the bases with the highest probability were AA**AAGGAG **(the bases in bold type had distinctly higher probabilities compared to the initial AA). This motif concurs with the consensus Shine-Dalgarno sequence for *B. subtilis *(AAAGGAGG) [46]. RBSFINDER identified the core AAGGAG motif in around 80% of the cases for Glimmer gene predictions and adjusted the start codon accordingly. Manual inspection and alignments to *B. subtilis *homologs were also used to determine the incidence of specific genes. During the gene-finding process, possible errors and frameshifts were detected by both visual inspection of the CDSs to look for interrupted or truncated genes and by deploying FrameD software [44]. Frameshifts were resolved by re-sequencing of PCR-amplified segments or subclones. After re-sequencing and manual editing a total of 27 frameshifts remain in the genome assembly (excluding those contained in the *IS3Bli1 *elements). It is not known at present whether these represent pseudogenes or instances of programmed translational frameshifting. The positions of rRNA operons in the genome assembly were confirmed by long-range PCR amplification using primers that annealed to genes flanking the rRNA genes. These PCR fragments were sequenced to high redundancy and the consensus sequences were manually inserted into the assembly. Among the seven rRNA operons, the nucleotide sequences of 16S and 23S genes are at least 99% identical, differing by only one to three nucleotides in pairwise comparisons. Protein-coding sequences were annotated in an automated fashion with the following software applications. Predicted proteins were compared to the nonredundant database PIR-NREF [47] and the *B. subtilis *genome [48] using BLASTP with a E-value threshold of 1 × 10^-5^. InterProScan was used to predict putative function [49]. The InterPro analysis included comparison to PFAM [50], TIGRFAM [51], Interpro [52] signal peptide prediction using SignalP [20] and transmembrane domain prediction using TMHMM [21]. These CDSs were assigned to functional categories based on the Cluster of Orthologous Groups (COG) database [53] with manual verification as described [54,55]. Phage gene boundaries were predicted using gene finding algorithms and by homology to known bacteriophage genes. Transfer RNA genes were identified using tRNAscan-SE [56]. *B. licheniformis *genes that shared significant homology with *B. subtilis *counterparts were named using the nomenclature in the SubtiList database [48] with updated gene names from the BSORF [57] and UniProt [58] databases.

### Comparative analyses

VisualGenome software (Rational Genomics) was used for comparisons of ortholog distribution among *B. licheniformis*, *B. subtilis *and *B. halodurans *genomes with precomputed BLAST results stored in a local database.

### Accession of genome sequence information

The GenBank accession number for the *B. licheniformis *ATCC 14580 genome is CP000002. An interactive web portal for viewing and searching the assembled genome based on the generic genome browser developed by Stein *et al. *[59] is available at [60].

## Figures and Tables

**Table 1:** Features of the *B. licheniformis *genome and comparison with genomes of other *Bacillus *species

Feature	*B. licheniformis*	B. subtilis*	*B. halodurans*^†^	*Oceanobacillus iheyensis*^‡^	*B. anthracis*^§^	*B. cereus*^¶^
Chromosome size (bp)	4,222,336	4,214,630	4,202,352	3,630,528	5,227,293	5,426,909
G+C content (mol%)	46.2	43.5	43.7	35.7	35.4	35.4
Protein coding sequences	4,208	4,106	4,066	3,496	5,508	5,366
Average length (bp)	873	896	879	883	800	835
Percent of coding region	86	87	85	85	84	84
Ribosomal RNA operons	7	10	8	7	11	13
Number of tRNAs	72	86	78	69	95	108
Phage-associated genes	71	268	42	27	62	124
Transposase genes of IS elements	10	0	93	14	18	10

*Kunst *et al. *[10]; ^†^Takami *et al. *[13]; ^‡^Takami *et al. *[16]; ^§^Read *et al. *[61]; ^¶^Ivanova *et al. *[62].

**Table 2:** Gene sequences corresponding to isochore peaks shown in Figure 3

Peak	Size (kb)	% G+C	*B. subtilis *orthologs	Annotation
1	3.2	28	No	ABC transporter, conserved hypothetical, and hypothetical genes
2	3.6	38	No	Conserved hypothetical and hypothetical genes
3	2.1	37	No	Conserved hypothetical and hypothetical genes
4	2.8	37	No	Hypothetical genes
5	2.7	37	No	Phosphotriesterase, conserved hypothetical genes
6	7.4	37	No	Type I restriction-modification system
7	3.5	38	No	Hypothetical genes
8	8.4	38	Partial	*yybO*, *pucR*, *pucH*, *yurH*, *ycbE*, *yjfA*, *rapG*, carbamate kinase, conserved hypothetical and hypothetical genes
9	10.1	36	No	SPP-1 like phage, conserved hypothetical and hypothetical genes
10	4.8	37	Yes	Hypothetical genes
11	3.0	33	No	Conserved hypothetical and hypothetical genes
12	4.3	34	No	Conserved hypothetical and hypothetical genes
13	2.2	34	No	Conserved hypothetical and hypothetical genes
14	5.4	36	Partial	Conserved hypothetical and hypothetical genes
15	4.4	35	No	Conserved hypothetical and hypothetical genes
16	4.6	33	No	ABC transporter and hypothetical genes
17	3.5	35	Partial	*comP*, *comX*, *comQ*, and *IS3Bli1*
18	6.8	37	No	*IS3Bli1*, conserved hypothetical and hypothetical genes
19	3.8	38	No	Phage w-105-like genes
20	6.8	35	Yes	*tagG *and *tagF *genes
21	3.2	34	No	Conserved hypothetical genes
22	1.7	34	No	Conserved hypothetical genes
23	1.6	37	No	Hypothetical genes
24	16.2	35	No	Type I restriction-modification system, conserved hypothetical and hypothetical genes
25	3.3	62	No	Hypothetical gene

## References

[B1] Alexander M (1977). Introduction to Soil Microbiology.

[B2] Eveleigh DE (1981). The microbial production of industrial chemicals.. Sci Am.

[B3] Erickson RJ, Schlesinger D (1976). Industrial applications of the bacilli: a review and prospectus.. In Microbiology.

[B4] Gherna R, Pienta P, Cote R (1989). American Type Culture Collection Catalogue of Bacteria and Phages.

[B5] Neyra C, Atkinson LA, Olubayi O, Sadasivan L, Zaurov D, Zappi E (1996). Novel microbial technologies for the enhancement of plant growth and biocontrol of fungal diseases in crops.. Cahiers Opt Méd.

[B6] Logan NA, Berkeley RCW, Berkeley RCW, Goodfellow M (1981). Classification and identification of the genus *Bacillus *using API tests.. In The Aerobic Endospore-Forming Bacteria: Classification and Identification.

[B7] O'Donnell AG, Norris JR, Berkeley RCW, Claus D, Kanero T, Logan NA, Nozaki R (1980). Characterization of *Bacillus subtilis*, *Bacillus pumilus*, *Bacillus licheniformis*, and *Bacillus amyloliquefaciens *by pyrolysis gas-liquid chromatography, deoxyribonucleic acid - deoxyribonucleic acid hybridization, biochemical tests, and API systems.. Int J Syst Bacteriol.

[B8] Xu D, Côté JC (2003). Phylogenetic relationships between *Bacillus *species and related genera inferred from comparison of 3' end 16S rDNA and 5' end 16S-23S ITS nucleotide sequences.. Int J Syst Evol Microbiol.

[B9] Lapidus A, Galleron N, Andersen JT, Jørgensen PL, Ehrlich SD, Sorokin A (2002). Co-linear scaffold of the *Bacillus licheniformis *and *Bacillus subtilis *genomes and its use to compare their competence genes.. FEMS Microbiol Lett.

[B10] Kunst F, Ogasawara N, Mozser I, Albertini AM, Alloni G, Azebedo V, Bertero MG, Bessieres P, Bolotin A, Borchert S (1997). The complete genome sequence of the gram-positive bacterium *Bacillus subtilis*.. Nature.

[B11] Christensen BB, Atlung T, Hansen FG (1999). DnaA boxes are important elements in setting the initiation mass of *Escherichia coli*.. J Bacteriol.

[B12] Majka J, Jakimowicz D, Messer W, Schrempf H, Lisowski M, Zakrzewska-Czerwiñska J (1999). Interactions of the *Streptomyces lividans *initiator protein DnaA with its target.. Eur J Biochem.

[B13] Takami H, Nakasone K, Takaki Y, Maeno G, Sasaki R, Masui N, Fuji F, Hirama C, Nakamura Y, Ogasawara N (2000). Complete genome sequence of the alkaliphilic bacterium *Bacillus halodurans *and genomic sequence comparison with *Bacillus subtilis*.. Nucleic Acids Res.

[B14] Farabaugh P (1996). Programmed translational frameshifting.. Microbiol Rev.

[B15] Bao Q, Tian Y, Li W, Xu Z, Xuan Z, Hu S, Dong W, Yang J, Chen Y, Xue Y (2002). A complete sequence of the *T. tengcongensis *genome.. Genome Res.

[B16] Takami H, Takaki Y, Uchiyama I (2002). Genome sequence of *Oceanobacillus iheyensis *isolated from the Iheya Ridge and its unexpected adaptive capabilities to extreme environments.. Nucleic Acids Res.

[B17] Takahashi S, Detrick S, Whiting AA, Blaschke-Bonkowksy AJ, Aoyagi Y, Adderson EE, Bohnsack JF (2002). Correlation of phylogenetic lineages of group B streptococci, identified by analysis of restriction-digestion patterns of genomic DNA, with *infB *alleles and mobile genetic elements.. J Infect Dis.

[B18] Smoot JC, Barbian KD, Van Gompel JJ, Smoot LM, Chaussee MS, Sylva GL, Sturdevant DE, Ricklefs SM, Porcella SF, Parkins LD (2002). Genome sequence and comparative microarray analysis of serotype M18 group A *Streptococcus *strains associated with acute rheumatic fever outbreaks.. Proc Natl Acad Sci USA.

[B19] Casjens S (2003). Prophages and bacterial genomics: What have we learned so far?. Mol Microbiol.

[B20] Nielsen H, Engelbrecht J, Brunak S, von Heijne G (1997). Identification of prokaryotic and eukaryotic signal peptides and prediction of their cleavage sites.. Protein Eng.

[B21] Krogh A, Larsson B, von Heijne G, Sonnhammer ELL (2000). Predicting transmembrane protein topology with a hidden Markov model: application to complete genomes.. J Mol Biol.

[B22] Sonnenshein AL (1993). Bacillus subtilis and Other Gram-Positive Bacteria: Biochemistry, Physiology, and Molecular Genetics.

[B23] Soong CL, Ogawa J, Honda M, Shimizu S (1999). Cyclic-imide hydrolyzing activity of D-hydantoinase from *Blastobacter *sp. Strain A17p-4.. Appl Environ Microbiol.

[B24] Gwinn DD, Thorne CB (1964). Transformation of *Bacillus licheniformis*.. J Bacteriol.

[B25] Thorne CB, Stull HB (1966). Factors affecting transformation of *Bacillus licheniformis*.. J Bacteriol.

[B26] Katz E, Demain AL (1977). The peptide antibiotics of *Bacillus*: chemistry, biogenesis, and possible functions.. Bacteriol Rev.

[B27] Ishihara H, Takoh M, Nishibayashi R, Sato A (2002). Distribution and variation of bacitracin synthetase gene sequences in laboratory stock strains of *Bacillus licheniformis*.. Curr Microbiol.

[B28] Grangemard I, Wallach J, Maget-Dana R, Peypoux F (2001). Lichenysin - a more efficient cation chelator than surfactin.. Appl Biochem Biotechnol.

[B29] Westers H, Dorenbos R, van Dijl JM, Kable J, Flanagan T, Devine KM, Jude F, Séror SJ, Beekman AC, Darmon E (2003). Genome engineering reveals large dispensable regions in *Bacillus subtilis*.. Mol Biol Evol.

[B30] Altena K, Guder A, Cramer C, Bierbaum G (2000). Biosynthesis of the lantibiotic mersacidin: organization of a type B lantibiotic gene cluster.. Appl Environ Microbiol.

[B31] Pag U, Sahl HG (2002). Multiple activities in lantibiotics - models for the design of novel antibiotics?. Curr Pharm Des.

[B32] Hoffmann A, Pag U, Wiedemann I, Sahl HG (2002). Combination of antibiotic mechanisms in lantibiotics.. Farmaco.

[B33] Kobayashi K, Ehrlich SD, Albertini A, Amati G, Andersen KK, Arnaud M, Asai K, Ashikaga S, Aymerch S, Bessieres P (2003). Essential genes in *Bacillus subtilis*.. Proc Natl Acad Sci USA.

[B34] Pedersen PB, Bjørnvad ME, Rasmussen MD, Petersen JN (2002). Cytotoxic potential of industrial strains of *Bacillus *sp.. Regul Toxicol Pharmacol.

[B35] Salkinoja-Salonen MS, Vuorio R, Andersson MA, Kämpfer P, Andersson MC, Honkanen-Buzalski T, Scoging AC (1999). Toxigenic strains of *Bacillus licheniformis *related to food poisoning.. Appl Environ Microbiol.

[B36] Agata N, Ohta M (2002). Identification and molecular characterization of the genetic locus for biosynthesis of the emetic toxin, cereulide, of *Bacillus cereus*.. Abstr Ann Meeting Am Soc Microbiol.

[B37] Manachini PL, Fortina MG, Levati L, Parini C (1998). Contribution to phenotypic and genotypic characterization of *Bacillus licheniformis *and description of new genomovars.. Syst Appl Microbiol.

[B38] De Clerck E, De Vos P (2004). Genotypic diversity among *Bacillus licheniformis *strains from various sources.. FEMS Microbiol Lett.

[B39] Wilson RK, Mardis ER, Birren B, Green ED, Meyers RM, Roskams J (1997). Shotgun sequencing.. In Genome Analysis: A Laboratory Manual.

[B40] Kim UJ, Shizuya H, de Jong PJ, Birren B, Simon MI (1992). Stable propagation of cosmid sized human DNA inserts in an F factor based vector.. Nucleic Acids Res.

[B41] Phred, Phrap, and Consed. http://www.phrap.org/phredphrapconsed.html.

[B42] Larsen TS, Krogh A (2003). EasyGene - a prokaryotic gene finder that ranks ORFs by statistical significance.. BMC Bioinformatics.

[B43] Delcher AL, Harmon D, Kasif S, White O, Salzberg SL (1999). Improved microbial gene identification with GLIMMER.. Nucleic Acids Res.

[B44] Schiex T, Gouzy J, Moisan A, de Oliveira Y (2003). FrameD: a flexible program for quality check and gene prediction in prokaryotic genomes and noisy matured eukaryotic sequences.. Nucleic Acids Res.

[B45] Suzek BE, Ermolaeva MD, Schreiber M, Salzberg SL (2001). A probabilistic, method for identifying start codons in bacterial genomes.. Bioinformatics.

[B46] Rocha EPC, Danchin A, Viari A (1999). Translation in *Bacillus subtilis*: roles and trends of initiation and termination, insights from a genome analysis.. Nucleic Acids Res.

[B47] Wu CH, Huang H, Arminski L, Castro-Alvear J, Chen Y, Hu ZZ, Ledley RS, Lewis KC, Mewes HW, Orcutt BC (2002). The Protein Information Resource: an integrated public resource of functional annotation of proteins.. Nucleic Acids Res.

[B48] SubtiList. http://genolist.pasteur.fr/SubtiList/genome.cgi.

[B49] Zdobnov EM, Apweiler R (2001). InterProScan - an integration platform for the signature-recognition methods in InterPro.. Bioinformatics.

[B50] Bateman A, Coin L, Durbin R, Finn RD, Hollich V, Griffiths-Jones S, Khanna A, Sonnhammer EL, Studholme DJ, Yeats C, Eddy SR (2004). The Pfam protein families database.. Nucleic Acids Res.

[B51] Haft DJ, Selengut JD, White O (2003). The TIGRFAMs database of protein families.. Nucleic Acids Res.

[B52] Apweiler R, Attwood TK, Bairock A, Bateman A, Birney E, Biswas M, Bucher P, Cerutti L, Corpet F, Croning MD (2001). The InterPro database, an integrated documentation resource for protein families, domains and functional sites.. Nucleic Acids Res.

[B53] COG. http://www.ncbi.nlm.nih.gov/COG.

[B54] Tatusov RL, Koonin EV, Lipman DJ (1997). A genomic perspective on protein families.. Science.

[B55] Koonin EV, Galperin MY (2002). Sequence - Evolution - Function: Computational Approaches in Comparative Genomics.

[B56] Lowe TM, Eddy SR (1997). tRNAscan-SE: a program for improved detection of transfer RNA genes in genomic sequence.. Nucleic Acids Res.

[B57] BSORF top page. http://bacillus.genome.jp.

[B58] bacsu. http://www.expasy.org/cgi-bin/lists?bacsu.txt.

[B59] Stein LD, Mungall C, Shu S, Caudy M, Mangone M, Day A, Nickerson E, Stajich JE, Harris TW, Arva A, Lewis S (2002). The generic genome browser: a building block for a model organism system database.. Genome Res.

[B60] BACSAP home. http://63.198.8.200.

[B61] Read TD, Peterson SN, Tourasse N, Baillie LW, Paulsen IT, Nelson KE, Tettelin H, Fouts DE, Eisen JA, Gill SR (2003). The genome sequence of *Bacillus anthracis *Ames and comparison to closely related bacteria.. Nature.

[B62] Ivanova N, Sorokin A, Anderson I, Galleron N, Candelon B, Kapatral V, Bhattacharyya A, Reznik G, Mikhailova N, Lapidus A (2003). Genome sequence of *Bacillus cereus *and comparative analysis with *Bacillus anthracis*.. Nature.

